# The role of community health workers in improving child health programmes in Mali

**DOI:** 10.1186/1472-698X-9-28

**Published:** 2009-11-10

**Authors:** Freddy Perez, Hamady Ba, Sayed G Dastagire, Mathias Altmann

**Affiliations:** 1 Institut de Santé Publique, d'Epidémiologie et de Développement (ISPED), Université Victor Segalen Bordeaux 2, 146 Rue Leo Saignat, 33076 Bordeaux, France; 2 Direction Régional de la Santé de Kayes (Conseiller technique), République de Mali

## Abstract

**Background:**

Mortality of children under the age of five remains one of the most important public health challenges in developing countries. In rural settings, the promotion of household and community health practices through community health workers (CHWs) is among the key strategies to improve child health. The objective of this study was to assess the performance of CHWs in the promotion of basic child heath services in rural Mali.

**Methods:**

A community-based cross-sectional survey was undertaken using multi-stage cluster sampling of wards and villages. Data was collected through questionnaires among 401 child-caregivers and registers of 72 CHWs.

**Results:**

Of 401 households suppose to receive a visit by a CHW, 219 (54.6%; confidence interval 95%; 49.6-59.5) had received at least one visit in the last three months before the survey. The mother is the most important caregiver (97%); high percentage being illiterate. Caregivers treat fever and diarrhoea with the correct regimen in 40% and 11% of cases respectively. Comparative analysis between households with and without CHW visits showed a positive influence of CHWs on family health practices: knowledge on the management of child fever (p = < 0.001), non-utilization of antibiotics in home treatment of diarrhoea (p = 0.003), presence of cloroquine in the household (p = 0.002), presence (p = 0.001) and use (p = < 0.001) of bed nets. A total of 27 (38%) CHWs had not received supervision at all, against 45 (63%) who have been followed regularly each month during the last six months.

**Conclusion:**

Continuous training, transport means, adequate supervision and motivation of CHWs through the introduction of financial incentives and remuneration are among key factors to improve the work of CHWs in rural communities. Poor performance of basic household health practices can be related to irregular supply of drugs and the need of appropriate follow-up by CHWs.

## Background

Every year, an estimated 11 million children in developing countries die before they reach their fifth birthday [1]. Seventy-three percent of these deaths are due to five childhood diseases occurring individually or in combination: diarrhoea, measles, acute respiratory infection, malaria and malnutrition [2]. These deaths could be prevented by available community-based interventions that are feasible to implement in resource-poor settings [3]. Nevertheless, the coverage of these effective interventions is low [4].

In Sub-Sahara Africa, an estimated 80% of acute febrile illness and death in infants and children under the age of five occur with little or no contact between household caregivers and professional health providers [5]. This is due to poor access to health services, insufficient resources at household level, poor knowledge and practice among caregivers and inadequate quality of heath facilities [6].

To expand the provision of health care, programmes involving community health workers (CHWs) have been implemented in resource-limited settings [7]. The World Health Organization (WHO) defines CHWs as members of the communities where they work, who should be selected by the communities, be supported by the health system but not necessarily a part of its organization, and have shorter training than professional workers [8]. In general terms, they receive a training programme that can be from one to two weeks to more than three months.

Since the 1980s, CHW programmes have been a cornerstone of primary health care based on the Alma-Ata declaration of 1978 [9]. There have been critical evaluations published concerning the role of CHWs, highlighting successes and problems (mainly related to sustainability and maintaining quality of care) as well as their potential in assisting in health and development issues [10,11]. CHWs have been considered as agents linked to behavioural change and as playing a key role in the extension of formal health services (7).

In 2002, UNICEF initiated the Accelerated Strategy of Child Survival and Development (ASCSD) in 11 central and western African countries, including Mali where data from 2006 report a mortality rate among infants and children under the age of five of 191 per 1000 births [12]. This is an integrated public health strategy where a package of essential child health services contributes to child survival and healthy growth. These services are provided at community level to rural populations who have difficult access to formal health facilities and a high child under-five mortality rate.

The Ministry of Health of Mali along with UNICEF have implemented this strategy composed of three cost-effective intervention packages at an operational level: Expanded Program of Immunization, basic maternal and newborn health services and the Integrated Management of Childhood illnesses (IMCI) [13]. The aim of the IMCI public health strategy is to reduce child mortality and improve child health and development and includes three components: improvement of case-management skills of health staff though the provision of locally adapted guidelines, improvement of health systems and improvement of household and community practices [14]. The objective of the household and community practice component of the IMCI strategy is to empower communities to address factors that affect child health, nutrition and development [15].

**Figure 1:**
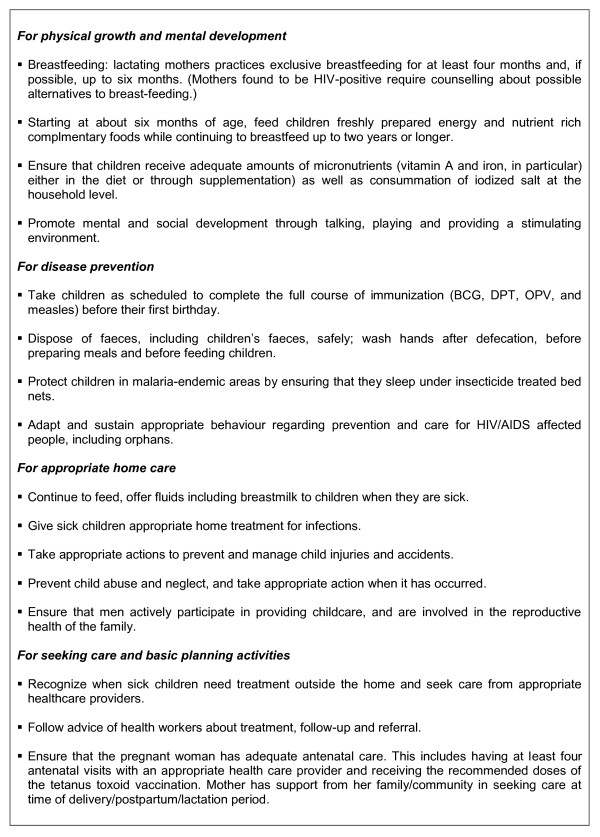
**List of key family practices for ensuring child survival (16)**.

The main component of the ASCSD public health intervention is the promotion of key family health practices which include physical growth and mental development, disease prevention, appropriate home care and care seeking behaviours. The effectiveness of these practices has been well documented in the literature [16] (Figure 1). The programme is characterized by a mix of household level outreach activities with community--level organizational tasks and social mobilization. These can be summarized in: 1) provision of health promotion and education of households on prevention and basic care management of child illness; 2) supporting the adoption of the key family health practices at the household level through regular home visits and; 3) facilitating out-reach and mobile activities under the responsibility of health centres.

For this, outreach CHWs are identified and trained for a period of five days to explain to their neighbours health prevention actions (personal hygiene, environmental sanitation, use of bed nets), basic child heath care management (for malaria, acute respiratory infection and diarrhoea), the importance of completing vaccination schemes among children and pregnant women, vitamin A supplementation, and periodic anthelminthic treatment. They are also given guidelines on how to coordinate the supply of household health kits and how to undertake periodic follow-up of households.

CHWs are provided with a bicycle as a means of transport to undertake periodic home visits. On average, it is planned that one CHW covers 245 inhabitants and visits 35 households, once per month. Thus, the CHW becomes a key actor of community IMCI and behaviour change communication in the community.

As part of this strategy, household basic health kits containing cloroquine and paracetamol and oral rehydration solution (ORS) salts are distributed through the referral health centre supply system during the CHW's monthly visits and outreach activities. Initially, kits were sponsored by UNICEF (cost $0.30 USD), then after, a full kit had a cost of $2.20 USD. In addition, insecticide-treated mosquito nets (ITNs) are distributed free of charge from the health care centre and during outreach activities to pregnant women and mothers who take their children for vaccination against preventable diseases such as measles and poliomyelitis

In Mali, no data is available at district level on the implementation of child health strategies at the community level The rural district of Djenné with a total population of 190 070 inhabitants [12] is among the first districts of Mali where different components of ASCSD, including the intervention of CHWs, have been piloted The objective of this study was to assess the performance of CHWs in the promotion of child health services at the household level in the district of Djenné, region of Mopti, Republic of Mali. The results of the study will provide data regarding ongoing child community health programmes; highlight improvements needed and set baseline information for periodic evaluations.

## Methods

### Study Design and population

A cross sectional descriptive study was undertaken. Qualitative and quantitative information regarding child health activities of CHWs was collected among caregivers at the household level. Sampling of households was done through a community-based multistage stratified, random cluster sampling procedure of households and villages in a rural district [17]. In order to obtain information on the acceptability and quality of the intervention being performed by CHWs, caregivers of children under five years of age were interviewed in their households. This study focused on the central issue of accessibility of rural households to basic care through a CHW.

The sample frame for this study considered the 33 592 households registered in the district. Sample size calculation was based on the assumption that approximately 50% of households have received a community health worker visit. Calculations were made to allow a precision of 5%, with an alpha type one error of 5%; which resulted in a minimum sample size 385 households to be surveyed. However, in order to overcome the problem of non-response, a sample size of 400 households was considered for this study.

From the total 167 existing villages of the district, 20 where randomly selected. Additionally, 20 households from each of the selected villages were randomly selected based on an existing numerated census list provided by each of the corresponding community leaders. Household sampling procedure was based on a primary and secondary stratification at district level. The primary stratification divided the district in three strata considering the distance of each "catchment area" of the district (group of villages that correspond to the coverage of a health facility) to the district referral hospital. Based on this approach, the district was divided into three strata. The first, second and third strata formed a circle with a radius of less than 20 kilometres, between 20 and 40 kilometres and more than 40 kilometres surrounding the district referral hospital. The total number of households to be selected from each of the three primary strata considered the total number of households in each of the primary strata, sample size and overall total number of households at district level. Two catchment areas were randomly selected from each of these three primary strata. Each of the three primary strata (which included to catchment areas and their corresponding villages), was divided in three secondary strata. This was based on the distance of each village to the corresponding community health centre. For this, distances of less then five kilometres, between five and ten kilometres and more than ten kilometres was considered. A total number of villages and their respective households for each of the nine secondary strata (three from each of the primary strata) were obtained. From the overall total of nine strata, 20 villages were selected based on a systematic random sampling method considering the number of households in each village. Of these 20 villages, 20 households were randomly selected.

Sampling of CHWs was determined by their availability during the visit to each of the selected villages but a minimum of 50% of the total assigned to the communities was planned. Their recruitment was done after explaining the aim of the study and having given consent.

### Data collection

Data collection for the study took place from April to May 2006. A questionnaire for caregivers of children under five years of age was designed composed of two main sections: 1) socio-demographic information of the child caregiver and; 2) knowledge and practices of household members concerning home-management of the sick infants and children under the age of five. The questions were written using a combination of multiple-choice and open-ended response formats [see Additional file 1]. The tool was pre-tested in a near by district and translated from French to Bambara and Peul.

An individual interview guide were designed to collect information from CHWs (n = 72) on socio-demographic variables, how they had been selected, training and supervision received, actual responsibilities, work load and perceptions concerning their motivation and future as a CHW. The monthly "register book" of CHWs' made it possible to record the adoption of the key family health practices at household level. Three possibilities are recorded: practice adopted correctly: « + » (household receive regular visits by CHWs and implementation of all health practices well adapted by the caregiver), incorrectly « 0 » (irregular visits by a CHW and not all household practices implemented) and practice not applicable: « - » (the household practice is not evaluated). For this study, data was drawn from registers concerning the month preceding the survey (April 2006).

The survey team was composed of four interviewers who were health promotion staff from the district hospital, had experience in field surveys and spoke the various Djenne dialects present in the district. In addition, four supervisors participated in the study which included one health staff from the district hospital and three co-authors of the study (BH, AM, GDS). The completed questionnaires were verified by the supervisors and data was entered into a specifically designed database throughout the survey. Prior to analysis, missing data was checked against the survey forms. Quantitative and categorical data was entered and analysed in EPI-INFO 2004 (US Centres for Disease Control and Prevention, Atlanta, GA). Caregiver and CHWs characteristics were described using median values and interquartile ranges (IQR) for continuous variables, and counts and percentages for categorical data. Differences of proportions were tested using an X^2 ^test.

### Ethical considerations

Authorisation to carry out the survey was obtained from the Ministry of Health and Child Welfare of Mali and the district health administrators. Verbal informed consent was obtained and confidentiality was assured for all the participants.

## Results

The study population included 401 caregivers (one additional household was interviewed and included in the study). The socio-demographic characteristics of caregivers of children under five are included in table 1. The profile of households is typical of a rural, West African population. The mother is the most important caregiver (97%); a high percentage being illiterate. The overall average number of children in each household was 3.3 and that of children under five was 1.5. Of the 219 households who received at least one visit from the CHW, 74 (33.7%; confidence interval [CI] 95% 28.1-41.2) declared preferring to go to the CHW before going to the health centre. Furthermore, 80 households (20%; CI 95% 16.8-25.1) declared having been involved one way or the other in the selection of the CHW.

**Table 1:** Socio-demographic characteristics of 401 households in the district of Djenné, Mali. Community health worker survey, April 2006 ±

Characteristics of the households	Number	%
**Caregiver in charge of children < 5 years of age**		
Mother	383	96.5
Grand-mother	14	3.5
**Level of education**		
Illiterate *	326	82.1
Literate in local language	43	10.8
Primary	26	6.5
Secondary	2	0.5
**Principal occupation**		
Housewife	387	98.5
Other	6	1.5
**Revenue generating activity**		
Yes	158	40.3
No	234	59.7
Geographical accessibility in raining season **		
No	261	65.0
Yes	140	35.0

± Based on the calculated sample size planned, one additional household was interviewed and included in the study

* Illiterate in local languages and French

** Months of the raining season which makes roads inaccessible: June to February

Of the 401 households suppose to receive a visit by a CHW, 219 (54.6%; CI 95% 49.6-59.5) had received at least one visit in the last three months and of these 162 (73.9%; CI 95% 67.8-79.7%) had received a visit the month before the survey (April, 2006). Regarding knowledge of the child caregiver on home management of child illness, 40% mentioned to have treated fever with the correct regimen: cloroquine plus paracetamol [18], 37% cloroquine alone and in a less percentage with paracetamol only (11%). Most caregivers report to have treated child diarrhoea with oxytetracycline (27%), traditional methods (preparations based on local plants) (24%) and others (including drugs bought in the informal system and not adapted to the child illness) (24%). In addition, correct knowledge of the use of cloroquine and paracetamol was 21% and 45% respectively (table 2).

**Table 2:** Household management of child illness (< 5 years) in the district of Djenné, Mali, Community health worker survey, April 2006 (n = 401).

Knowledge/practice of the caregiver regarding specific illnesses	Number	%	CI 95%
Management of fever *:			
Chloroquine	149	37.0	32.4 -- 42.1
Paracetamol	42	11.0	7.7 -- 14.0
Chloroquine + Paracetamol	162	40.0	35.6 -- 45.4
other	48	12.0	9.0 -- 15.7
Treatment of diarrhoea* :			
ORS ±	43	10.7	8.0 -- 14.3
SSS ±	5	1.2	0.5 -- 3.1
Oxytetracycline	111	27.7	23.4 -- 32.4
Other**	96	23.9	19.9 -- 28.5
Traditional**	96	23.9	19.9 -- 28.5
Other over counter drugs**	18	4.5	2.8 -- 7.1
Doing nothing	29	7.2	5.0 -- 10.3
**Correct knowledge of dosage**			
Chloroquine	85	21.2	17.4 -- 25.6
Paracetamol	179	44.6	39.7 -- 49.7
**Possession of « household kit »**			
Yes	47	11.8	8.9 -- 15.5
No	352	88.2	84.6 -- 91.2
**Presence of drugs in the household**			
Chloroquine	73	18.2	14.6 -- 22.4
Paracetamol	42	10.5	7.7 -- 14.0
ORS	3	0.7	0.2 -- 2.4
Over counter drugs	18	4.5	2.8 -- 7.1
**Presence/use of bed net**			
Presence	315	78.6	74.1 -- 82.4
Being used	216	57.8	52.8 -- 62.8
Impregnated	64	16.6	12.9 -- 20.3
**Presence of iodized salt in the household**			
Iodized salt	376	98.7	96.8 -- 99.5

C.I. = confidence interval

± ORS = oral rehydration therapy; SSS = home-made salt/sugar solution

* Correct answer: for fever: chloroquine plus paracetamol, for diarrhoea SRO (26)

** Other: other drugs bought in the formal market, example: amoxiciline, drugs for a cold; traditional: beavers based on local plants; over the counter drugs: drugs acquired through the informal system.

Results of the observed presence of supplies in households for home management of child under-five illness showed that 11.8% possessed a household kit, presence of drugs in the household was less than 20% for cloroquine and paracetamol and almost null for oral rehydration salt (ORS). Seventy-nine percent of the visited households had a bed net, of these, 16.6% were impregnated and through direct observation, 58% were registered as being used. Finally, a high percentage of households had iodized salt (98.7%) (table 2).

Based on district health registers, of a total of 99 CHWs from the twenty randomly selected villages, 72 (78%) were present during the survey. Male and female were of equal number, the medium age was 42 years old (range 18 to 83) and their main occupation being agriculture (49%) and house chorus (44%). Of these, 51.4% had some primary school and 22% did not know how to read and write (table 3). Based on reporting by CHWs, their workload represents on average of ten hours per month, which is done in three to seven days. One-third of CHWs declared that their work-load is too intensive. Follow-up of CHWs which was to be conducted on a monthly base was under the responsibility of a local non-governmental organization. A total of 27 (38%) CHWs had not received supervision at all, against 45 (63%) who have been followed regularly each month during the last six months. Very few follow up supervisions (17% of CHWs) had been carried out by health staff of the community health centres and no supervision had been undertaken by health staff of the district hospital as initially planned. In addition, 81% (n = 58) of CHWs stated that the duration of the training received was to short to enable them to have a full understanding of the practices they are suppose to undertake and that they encounter a lack of support from supervisors.

**Table 3:** Socio-demographic characteristics of community health workers in the district of Djenné, Mali. Community health worker survey, April 2006 (n = 72).

Characteristics of the community health worker	Number	%
**Sex**		
Male	35	48.6
Female	37	51.4
**Main activity**		
Housework	31	44.3
Agriculture	34	48.6
other	5	7.1
**Level of education**		
Illiterate*	16	22.2
Literate in local language	17	23.6
Primary	37	51.4
Secondary	2	2.8
**Capable to read and write**		
In French	33	45.8
In Arabic	3	4.3
In local language	19	26.4
Doesn't know how to read nor write	17	23.6
**Revenue generating activity**		
Yes	39	54.2
No	33	45.8
**Has received initial training**		
March 2003	26	36.1
September 2005	46	63.9

* Illiterate in local languages and French

Data collected through the "register books" of 71 CHWs for the month preceding the survey showed that 54 registers were completed with information on all key family health practices they are suppose to implement and/or follow-up (Box 1). The quality of implementation of these practices by households based on registered observations undertaken by CHWs showed that 59% were well adopted, 16% poorly adopted and 19% not applicable. The main topics discussed by the CHWs during their home visits sessions were: IMCI 47.0% [CI: 40.3 -- 53.9], EPI 31.5%, [CI: 25.4 -- 38.1] and basic maternal and newborn care 33.3% [CI: 27.1 -- 40.0].

Of the total of 401 households, 78 (19.5%) were found to have no link to a CHW (even though this had been programmed). With this outcome, a comparative analysis between households with and without CHWs was undertaken. Results showed no statistically significant difference concerning socio-demographic characteristics (table 4). When compared on knowledge and practice, a positive influence of CHWs on specific essential family health practices by the households was found, namely knowledge on the management of child fever (p = < 0.001), non-utilization of antibiotics in home treatment of diarrhoea (p = 0.003), presence of cloroquine in the household (p = 0.002), presence (p = 0.001) and use (p = < 0.001) of bed net and, utilization of iodized salt (p = 0.05) (table 5).

**Table 4:** Comparison of qualitative variables between households with and without a community health worker in the district of Djenné, Mali.

	Households without support from a community health worker † (n = 78)*		Households with support from a community health worker (n = 323)*		P value ±
			
Characteristics	Number households	%	Number household	%	
**Caregiver in charge of child < 5**					0.589
Mother	73	94.8	310	96.9	
Grand-mother	4	5.2	10	3.1	
**Level of education**					0.577
Illiterate	68	87.2	258	80.9	
Literate in local language	6	7.7	37	11.6	
Primary	4	5.1	22	6.9	
Secondary	0	0.0	2	0.6	
**Principal occupation**					0.842
Housework	74	100.0	313	98.1	
Other	0	0.0	6	1.9	
**Revenue generating activity**					0.304
Yes	35	45.5	123	39.0	
No	42	54.5	192	61.0	
Geographical accessibility in raining season **					0.39
Floodable	24	30.8	116	35.9	
Not floodable	54	69.2	207	65.1	

Community health worker survey, April 2006

† These are households that were found to have no CHW support even though this was planned. They were used as a control group. * Totals may not sum to n = 78 and n = 323 due to missing observations; ± missing data not included in the calculation; ** Months of the raining season which makes roads inaccessible: June to February

**Table 5:** Comparison of knowledge and practice between households with and without community health workers in the district of Djenné, Mali.

Knowledge and health practices of households	Household without support from a community heath worker † n = 78 *		Household with support from a community heath worker n = 323*		P value ±
	**Number**	**%**	**Number**	**%**	
			
Correct Treatment of child fever^a^	60	76.9	293	90.7	**< 0.001**
Correct Treatment of child diarrhoea	5	6.4	42	13.0	0.104
Use of oxytetracycline	32	41.0	79	24.5	**0.003**
Knowledge of drug doses					
- Correct for chloroquine	16	20.5	69	21.6	0.839
- Correct for paracetamol	34	43.6	145	44.9	0.835
Possession of household health kit					
Yes	10	12.8	37	11.5	0.750
Presence of paracetamol in house					
Yes	5	6.4	37	11.5	0.192
Presence of chloroquine in house					
Yes	5	6.4	68	21.1	**0.002**
Presence of oral rehydration solutions in household					
Yes	0	0	3	0.9	0.522
Presence of over the counter drugs	2	2.6	16	5.0	0.542
Bed net					
- Presence	51	65.4	264	81.7	**0.001**
- Being used (n = 264)	25	51.0	191	79.9	**< 0.001**
- Impregnated (n = 264)	10	20.4	54	21.5	0.863
Iodized salt	71	95.9	305	99.3	**0.050**

Community health worker survey, April 2006

† These are households that were found to have no CHW support even though this was planned. They were used as a control group

* Totals may not sum to n = 78 and n = 323 due to missing observations; ± missing data not included in the calculation;

^a ^Correct = cloroquine plus paracetamol

## Discussion

The Millennium Development Goal of reducing child mortality by two-thirds by the year 2015 will not be achieved unless there is a massive increase in available community-based interventions in resource-poor settings. It has been recognized that an improvement in the quality of health facilities is not itself sufficient to reduce childhood morbidity and mortality because many caregivers do not seek treatment for their children at health facilities.

Even though there have been studies that have shown a reduction in child mortality linked to community health workers [7], to our knowledge, our study is among the few to provide results of child health community programmes in West Africa [19,20]. In this rural context, the mother has the primary role of caring for the child. Her principle occupation is being a housewife and has a minimum level of education. These socio-demographic characteristics are similar to those of caregivers in other settings [21]. Huicho and colleagues showed that for the community component of the IMCI strategy implementation in Peru, caregivers' low level of education needs to be considered as a potential constraint to the implementation of child survival interventions and coverage of community IMCI [22]. In this sense, there is a need to use communication strategies and tools adapted to the education level of the population to improve their knowledge of child health that can be put into practice, encourage health-seeking behaviour, and improve compliance with advice and treatment from health professionals. Studies have shown that the use of flipcharts, picture card techniques and role playing have been effective job aids for CHWs in developing countries [23].

Our study has evaluated knowledge and practice concerning home management of fever and diarrhoea among infants and children under the age of five as a proxy indicator of the performance of CHWs at household level. Results indicate that correct management of fever has been relatively good (40%). In contrast, management of diarrhoea is poor. These results show the importance of continuous provision of appropriate advice to caregivers due to the need of greater adherence to simple clinical practice guidelines and the development of sound communication strategies involving communities and households. This can only happen if there are also available drugs and supplies at the community level.

Overall, we documented a high percentage of incomplete kits among the households surveyed (89%), as example, the presence of chloroquine in only 18% of cases, the presence of paracetamol in only 11% of cases and almost no ORS. This highlights that the notion of "having a complete kit" has not been give a priority and not applied. This could be due to the provider of the kit not continuing to subsidize the sale and cost of these "household kit". Noteworthy, only 4% of households are ready to pay the actual price of the kit (2.18 US$). The non-availability of "household kits" in the villages, and the high cost of theses kits can explain the reason why rural communities use "over the counter drug sellers" present in the villages.

During the period of this study, cloroquine was the first-line recommended drug for the treatment of uncomplicated malaria in Mali. Recent reports of the emergence of *Plasmodium falciparum *resistance to cloroquine in different endemic settings of the country [24] have compelled the Ministry of Health of Mali to change to artemisin-based combination therapy (ACT) for first-line treatment of uncomplicated malaria, in line with the recommendation of the World Health Organization [25]. Local studies are needed to identify the most appropriate strategies for home management of malaria including the distribution of ACT at the community level. This will require the provision of prompt and effective treatment of malaria as well as follow-up on the possibility of over-prescription of the drug [26].

Results show that caregivers still give their children unnecessary antibiotics for diarrhoea. A common practice in rural areas of Mali is the use of oxytetracycline, called in local language "*Kunbiléni*" for treatment of diarrhoea and sold by over the counter drug sellers who visit the villages [27]. This drug is not recommended for children under the age of five due to its association with dental staining and interference with bone growth [28]. These findings have implications for education efforts to increase appropriate treatment of diarrhoea and malaria and establish systems to maintain an adequate supply of essential equipment and reinforce partnership between health facilities and the communities they serve. This will contribute to decrease inappropriate use of antibiotics and thus hopefully limit the development of antibiotic resistance.

The use of insecticide-treated mosquito nets (ITNs) has been shown to reduce malaria transmission, clinical disease and all-cause under-five mortality [29]. A relatively high percentage of households in this study possessed a bed net (79%). However, of these, only 20% were found to be insecticide-treated mosquito nets (ITNs) and overall, through direct-observation by CHWs, 68% were being used. Even though untreated nets have been reported to provide some protection [30], the full benefits are only realized when nets are regularly retreated with insecticide [31]. The potential existence of factors present at community level such as cost and limited availability of both nets and insecticides, which have been reported to cause low use of ITNs, will need to be explored [32]. Improvements in net delivery are an essential pre-requisite to achieve high rates of ITN usage. It is necessary to continue training CHWs and sensitizing populations at the community level on the importance of the utilization of bed nets all year round. The CHW has a role to play in the campaign of re-impregnation of old bed nets and help in the distribution process of new bed nets.

Selection criteria for community health workers have been reported as a key factor in the performance of their activities [33]. In this rural context, male and female CHWs were of equal number, more than half had a primary school level education, agriculture and house chorus being the main occupation. These characteristics are in agreement with other publications [34]. In the district of Djenné, recruitment of CHWs was done mainly through local and national health authorities. Various authors have highlighted the need for recruiting CHWs from communities they serve and the importance of community participation in the selection and monitoring of community health workers [35]. A key strategy in this setting will be to involve the community in the selection and monitoring of CHWs.

Overall results of the household survey showed that CHWs had covered only 40% of the houses planned and take on average nearly 10 hours a month for these health activities. However, they indicated having difficulty to undertake home visits and consider they have work overload. In the literature, the definition of a CHW states that they should work part-time enabling them therefore to subsist by performing agriculture or other work and possibly by receiving a subsidy from either the local community or the national health services [8]. There are varied experiences concerning the conditions in the employment of CHWs. In several programmes they are employed on a voluntary basis [36,37], as in this setting, or as a regular employee with a fixed monthly salary [38]. Local health authorities and community representatives will need to clarify how CHWs will be employed as this factor will have an impact on their motivation to perform their tasks and the feasibility of sustainable community health activities in this context.

Thirty-eight percent of CHWs in our study did not receive any supervision during the last six-months. Although it is widely considered important that the supervision of CHWs be undertaken by both the community and professional health workers, this is often not the case. Barriers such as social distance between health personnel, CHWs and communities due to poor understanding of community participation have been highlighted [39]. Studies have shown that when supervision of the village-level health services was vigorous and extra resources were directed to primary health care, child mortality improved [40]. Community health interventions require a minimum of supervision to CHWs that will contribute to the quality of basic care provided at district and local levels.

The magnitude of differentials between households with CHWs and those without showed that in general terms, deployment of CHWs in the community was associated with households' better managing child illness, specifically treatment of child fever and non-use of antibiotics in home treatment of diarrhoea. The influence of the presence of CHWs in preventive tasks also resulted in a significantly higher presence and use of bed nets for the prevention of malaria. Furthermore, households with CHWs use more frequently the drugs indicated for management of child fever (chloroquine and/or paracetamol) than the households not having community liaison. However, no significant difference was found among the two groups regarding the correct dosing of these two drugs. The low level of literacy among CHWs as well as among the parent or caregiver in charge of children can be a reason why these populations have difficulty in mastering drug dosing. Here again, considering the importance of home-management in the IMCI strategy, child health information and education to households through CHWs and continuous supervision of the quality of the work of CHWs is needed

Potential limitations to the survey need to be acknowledged. Among them its cross-sectional design only allows to evaluate the association between the presence of a CHW and the management of child illness by caregivers in a one-time period rather than the management over time. Measurement of exposure to CHWs at household level was subject to recall from the caregiver. Also, the CHWs register was the only written document used in this study. Data from other sources such as the pregnancy register (antenatal and post-natal follow-up services) as well as the child health card (vaccination and child illness) would have been useful to compare our results. As so, the estimated results of the interventions should be interpreted with caution. To consider that even though it was possible to compare households who had and had not received support from a CHW, it is possible that these two groups interacted socially in terms of knowledge of household practices. Finally, the performance of CHWs was not evaluated under direct observation. Even though some characteristics of caregivers and CHWs may be specific to rural Mali, common lessons can be learned and generalized on a broader scale, specially given the widespread prevalence of child morbidity and mortality in Sub-Sahara Africa.

This study gives the possibility to better understand the contribution of CHWs concerning knowledge and practice of child illness at the house-hold level and the potential barriers and facilitating factors linked to this. Compared with health facilities, CHWs are geographically closer and available when health facilities are closed. As so, they can help ensure that home-based treatment for children is appropriate.

## Conclusion

Reinforcing the role of CHWs can facilitate the improvement of child health when strategies such as upgrading existing lower-level facilities, improving and building referral systems, training and supervision are considered. There is few evidence-based data on community interventions and the impact of community health workers in improving child health. Operational research in this field is still needed since it is likely that in the foreseeable future, community health workers could contribute to the uptake and adequate coverage of child health services that are needed to fulfil the MDGs in mother and child health in many countries in sub-Saharan Africa and elsewhere.

## Competing interests

The authors declare that they have no competing interests.

## Authors' contributions

FP: participated in the design, supervised study implementation, wrote the manuscript. HB, SGD, MA: participated in the design, performed analysis and interpretation of the data, coordinated overall survey implementation and data collection and revised the paper critically for intellectual content. All authors read and approved the final manuscript.

## Pre-publication history

The pre-publication history for this paper can be accessed here:

http://www.biomedcentral.com/1472-698X/9/28/prepub

## Supplementary Material

Additional file 1 **QUESTIONNAIRE D'ENQUÊTE DE SATISFACTION, DJENNE, 2006**. The questionnaire for caregivers of children under five years of age composed of two main sections: 1) socio-demographic information of the child caregiver and; 2) knowledge and practices of household members concerning home-management of the sick infants and children under the age of five. Click here for file
