# Supplementary material for: The role of community health workers in improving child health programmes in Mali
Source: BMC Int Health Hum Rights. 2009 Nov 10;9:28. doi: 10.1186/1472-698X-9-28 (PMC2782322; doi:10.1186/1472-698X-9-28)
Supplement: Additional file 1 — QUESTIONNAIRE D'ENQUÊTE DE SATISFACTION, DJENNE, 2006. The questionnaire for caregivers of children under five years of age composed of two main sections: 1) socio-demographic information of the child caregiver and; 2) knowledge and practices of household members concerning home-management of the sick infants and children under the age of five. [file 1472-698X-9-28-S1.doc]

**ANNEXE I**

N° ID Fiche : /__/__/__/

N° ID Relais : /___/___/

**QUESTIONNAIRE D’ENQUÊTE DE SATISFACTION, DJENNE, 2006**

Les questions seront posées en face à face de manière semi-ouverte

Nom enquêteur : _____________________

**I) Identification de l’enquêtée**

1. Aire de santé : _______________________
2. Nom du village : ____________________________
3. caractéristique géographique du village :……………………….……………………………………………………………………………………….…/___/

□ Inondé

□ Exondé

1. distance village / Centre de santé (en km) : …..………………………………………………………………………………………….……………/___/___/
2. Statut :………………………………………………………………………………………………………………………………..………………..……./___/

□ Mère

□ Autre_________________

1. Niveau d’instruction :………………………………….…………………………………………………………………………..……………………..…/___/

□ Analphabète

□ Alphabétisé autre langue : _________________

□ Primaire

□ Secondaire

□ Supérieur

1. Principale occupation :……………………….…………………………………………………………………………………..…………………………/___/

□ Ménage

□ Commerce

□ Fonctionnaire

□ Agriculture

□ Pêche

□ Elevage

□ Autre : ____________

1. Activité génératrice de revenu ? :……………………….………………………………………………………………………………..…………………/___/

__________________________

1. Nombre total d’enfant vivant:…………………………………………………………………………………………………….…………..………. /___/___/
2. Nombre d’enfant de moins de 5 ans vivant :…………………………………………………………………………………………….…….……. /___/___/
3. âge du dernier enfant (en mois) vivant :……………………………………………………………………………………………………….…….. /___/___/

# II) Satisfaction de l’enquêté / relais

1. Que signifie pour vous le mot « relais communautaire » ? ……………………………………………………………………………..…………...…../___/

*Donner la signification du relais*

1. Avez vous été impliquée dans le choix des relais de votre village ? ……………………………………………………………………………………../___/

□ Oui

□ Non

1. Quels sont les nom et prénom du relais qui s’occupe de votre ménage ?
2. Sexe du relais qui s’occupe de votre ménage : ………………………..…………………………………………………………………………….........../___/

□ Masculin

□ Féminin

1. Etes vous d’accord sur le choix de votre relais ? …………………………………………………………………………………………….....…………/___/

□ Oui

□ Non

1. A il visité au moins une fois votre ménage ? ………………….....……………………………………………………………………………..…………/___/

□ Oui

□ Non

**(Si la réponse est non, passez à la question 29)**

1. Combien de fois avez-vous reçu la visite du relais le mois dernier..…………………………………………………………………………..…….... /___/___/
2. Préférez vous consulter le relais ou aller au centre de santé ?……………………………………………………………………………………….….…../___/

□ Relais, pourquoi ___________________________________________________

□ Centre de santé ____________________________________________________

1. Avez vous fait recours au relais pour les problèmes de santé ? …………………………………………………………………………………..…….…../___/

□ Oui

□ Non ; Pourquoi : ___________________________________________

**(Si non, passer à la question 24)**

1. Pour quels problèmes de santé ? …………………………………………………………………………………………………………………..…….…./___/

# □ Fièvre

□ Diarrhée

# □ Toux ou difficulté respiratoire

□ Grossesse

□ Vaccination enfant

□ Autres à préciser____________________

1. Combien de fois avez vous fait appel au relais le mois dernier ? … ………………………………………………………………………………/___/___/
2. A-t-il répondu à votre dernier appel ? ………………………………………………………………………………………………………………..../___/

# □ Le même jour

□ Le lendemain

□ Pas du tout

1. thèmes abordés lors des VAD : …………………………………………………………………………………………………………………………/___/

1. Le relais vous a-t-il déjà conseillé d’aller au centre de santé ? ……................................................................................................................................/___/

# □ Oui

# □ Non

1. Quel rôle a joué le relais pour la santé de votre ménage ?
2. Le relais a-t-il déjà pratiqué des soins sur un membre du ménage ? ……………………………………………………………………………..……......./___/

□ Oui ; lequel : ____________________

□ Non

1. Recevez vous des médicaments de la part du relais en dehors des campagnes de masse ?...................................................................................................................................................................................................................................../___/

□ Oui, lequel : ____________________

□ Non

1. Avez-vous eu au moins une fois le « kit ménage » ? ……………………………………………………………………………………………....…...……/___/

# □ Oui

□ Non

1. Quels sont les médicaments présents dans le foyer *(à vérifier)*………………………………………………………………………………………………../___/

□ Paracétamol *(quantité)*…………..

□ Chloroquine *(quantité)*…………..

□ SRO *(quantité)*…………..

□ Autre a préciser_________________________

1. Jusqu'à quel prix etes vous prêt a payer le kit ménage……………………..…………………………………………………………………………………../___/

*(L’enquêteur doit définir le kit, les maladies que les médicaments traitent et pendant combien de temps)*

1. Le relais explique-t-il l’utilisation/importance du kit ménage …………………………………………………………………………………………………/___/

# □ Oui

□ Non

□ Non applicable (pour ménage sans relais)

**III) Connaissances et pratiques des ménages**

1. Avec quel(s) médicament(s) traitez vous un enfant qui a un corps chaud ?............................................................................................................................/___/

□ Chloroquine

# □ Paracétamol

# □ Autres

□ Ne sait pas

1. Comment traitez vous votre dernier enfant avec la chloroquine ?

__________________________________________________________________

1. Comment traitez vous votre enfant quand il a la diarrhée ? ……………………………………………………………………………………………...…/___/

□ SRO

# □ SSS

□ Arrêt de l’alimentation (liquide et/ou solide)

# □ Ne fait rien

□ Autres____________________

1. Quelle quantité de paracétamol donnez vous à votre dernier enfant ? ____________________________________________________________________
2. Avez-vous une moustiquaire ? ……………..……………………………………………………………………………………………………………...…/___/

□ Oui

□ Non

1. A-t-elle été imprégnée dans les 6 derniers mois ? ……………..…………………………………………………………………………………..……...…/___/

□ Oui

□ Non

1. Est-elle attachée au dessus du lit de l’enfant ? *(à vérifier)*….………………………………………………………………………………………..………/___/

□ Oui

□ Non

1. Résultat du test à l’iodation du sel est il positif ? ………………………………………………………………………………………………………….…/___/

□ Oui

□ Non

# IV) Perspectives

1. Que conseillez vous pour améliorer les activités du relais ?
2. Que proposez vous d’autre que les relais pour améliorer la santé des enfants de moins de 5 ans et de la femme enceinte ?
